# Lead concentrations in antlers of European roe deer (*Capreolus capreolus*) from an agricultural area in Northern Germany over a 119-year period—a historical biomonitoring study

**DOI:** 10.1007/s11356-021-14538-6

**Published:** 2021-05-27

**Authors:** Catharina Ludolphy, Uwe Kierdorf, Horst Kierdorf

**Affiliations:** Department of Biology, University of Hildesheim, Universitätsplatz 1, 31141 Hildesheim, Germany

**Keywords:** Bioindication, Deer, Environmental pollution, Germany, Herbivores, Leaded gasoline

## Abstract

We analyzed the lead content in antlers of 90 adult European roe bucks (*Capreolus capreolus*) that had been culled between 1901 and 2019 in an agricultural-dominated hunting district in Lower Saxony (Northern Germany). Antler lead values ranged between 0.2 and 10.9 mg/kg dry weight. Median lead concentration was highest after World War II, during a period (1956–1984) of rapidly increasing mass motorization and use of leaded gasoline. Lead levels in antlers decreased markedly after the phase-out of leaded gasoline, but high values were still found in some recently collected antlers. This could indicate persistent lead pollution from former use of lead additives to gasoline, other traffic-related sources, or from agricultural sources (e.g., sewage sludge, fertilizers). This study highlights the suitability of analyzing roe deer antlers for the historical monitoring of changing lead levels in the environment. By collecting antlers and providing them for study, local hunters can significantly contribute to environmental surveillance and the monitoring of environmental pollution by bone-seeking contaminants.

## Introduction

Lead (Pb) is a metal that has no physiological function and is toxic even at low concentrations (Ewers and Schlipköter 1991; Pattee and Pain 2003; Ma 2011; Caito et al. 2017; Maret 2017). Various mammalian organs and organ systems are affected by lead toxicity, the most severe impacts concerning the nervous and hematopoietic systems and the kidneys (Ma 2011; Caito et al. 2017; Maret 2017). The developing brain is particularly susceptible to the toxic effects of lead, and therefore, lead neurotoxicity is an issue of special concern in children (Lidsky and Schneider 2003; Caito et al. 2017).

Lead mining dates back to at least the 4th millennium BC, and Pb mining and smelting activities were widespread in ancient Greek and Roman societies (Retief and Cilliers 2005). The emissions from ancient Greek and Roman lead and silver mining and smelting activities caused widespread lead pollution in the Northern Hemisphere that has been traced both in Greenland ice cores (Hong et al. 1994) and in European bogs (Shotyk 1998).

Compared to the pre-industrial period, the production and anthropogenic discharge of lead dramatically increased during the industrial age, with a particularly steep rise in the second half of the twentieth century (Cullen and McAlister 2017). Thus, global refined lead production amounted to 11.76 million tonnes in 2019 (U.S. Geological Survey 2020). Anthropogenic lead emissions to the atmosphere during the mid-1990s, approximately 120,000 tonnes/year, by far exceeded median fluxes from natural sources, estimated at 12,000 tonnes/year (Cullen and McAlister 2017). The drastically increased discharge of lead from human activities led to widespread lead pollution of the environment on a global scale, with the highest levels of contamination near urban-industrial areas (Nriagu 1990; Hernberg 2000).

Major anthropogenic sources of lead release to the environment include the combustion of fossil fuels for electricity and heat production, the mining and smelting of lead and other metal ores, iron and steel production, cement production, the use of lead-containing products (like batteries, ammunition, and paint), waste disposal, and vehicular traffic (Stroud 2015; Cullen and McAlister 2017; Baranowska-Bosiacka et al. 2019; Pain et al. 2019a, 2019b). Emissions from the latter source were the dominant cause of global lead pollution during much of the twentieth century, resulting in serious impacts on human and environmental health (Cullen and McAlister 2017; Filella and Bonet 2017). Lead emissions from vehicular traffic were largely caused by the use of alkyl-lead additives (mostly tetraethyl lead, TEL) as antiknock agents in gasoline (Stroud 2015; Filella and Bonet 2017). The inorganic lead derived from the organolead additives is highly persistent in the environment and, despite the more recent phasing out of leaded gasoline, high concentrations of legacy lead are therefore present in soils (Filella and Bonet 2017).

Commercial production of TEL started in 1923 and worldwide increased dramatically during the following decades (Hernberg 2000). The struggle to remove lead from gasoline extended over many years (Hernberg 2000; Needleman 2000; von Storch et al. 2003) and was achieved against strong resistance from industry and industry-funded researchers that denied or tried to downplay the public health risks of using leaded gasoline (Hernberg 2000; Needleman 2000). In the Federal Republic of Germany (FRG), the production and importation of gasoline containing more than 0.4 g Pb/liter were prohibited in 1972. Up to then, the usual lead content of gasoline had been 0.6 g/liter. In 1976, the permissible concentration of lead in gasoline was further reduced to 0.15 g/liter (von Storch et al. 2003). Unleaded gasoline (tolerable lead content of 0.013 g/liter) was introduced in the FRG in October 1984, regular leaded gasoline was banned in 1988 (while leaded premium gasoline was still allowed), and since 1997, leaded gasoline is no longer sold in Germany. The lowering of the lead content of gasoline and the subsequent phase-out of leaded gasoline, along with measures reducing lead release from other sources, caused a drastic reduction of anthropogenic lead emissions to the atmosphere, from 16,446 tonnes in 1975 (values for FRG and German Democratic Republic combined, von Storch et al. 2003) to 716 tonnes in 1995 in reunified Germany (European Monitoring and Evaluation Programme (EMEP) - Centre on Emission Inventories and Projections 2021). Since then, lead emissions in Germany further declined to 207 tonnes in 2018 (EMEP. Centre on Emission Inventories and Projections 2021). Currently, industry is the main lead emission source, followed by road traffic, the latter now primarily due to tire wear and brake abrasion (McKenzie et al. 2009; Grigoratos and Martini 2015; Adamiec et al. 2016; Adamiec 2017; Bourliva et al. 2018). Recent studies on lead exposure of biota and humans demonstrate that lead contamination of the environment remains an issue of high concern (Müller-Graf et al. 2017; Stokke et al. 2017; Gerofke et al. 2018, 2019; Martin et al. 2019; Pain et al. 2019a; Helander et al. 2019; Taggart et al. 2020; Lermen et al. 2021).

Lead uptake into the mammalian body occurs primarily via the gastrointestinal tract and the respiratory system. Upon absorption, lead rapidly enters the bloodstream and is transported to different tissues (Ma 2011; Caito et al. 2017). In the body, lead accumulates predominantly in mineralized tissues, and approximately 95% of the body burden of lead in adult humans (75% in children) is present in bones and teeth (Caito et al. 2017). Lead is taken up into the bone mineral (carbonated hydroxyapatite), where the Pb^2+^ ion is incorporated at Ca^2+^ sites during bone formation, while after the stop of crystal growth, it can replace Ca^2+^ by ion substitution (Pemmer et al. 2013). Lead stored in the skeleton is considered a biological marker of long-term exposure; however, under certain physiological and pathological conditions associated with increased bone turnover, skeletally stored lead can be mobilized and re-enter the bloodstream (Caito et al. 2017).

Several studies have demonstrated that antlers are well suited to monitor lead pollution of the environment (Sawicka-Kapusta 1979; Medvedev 1995; Kierdorf and Kierdorf, 1999, 2000a,b, 2002a,b, 2003, 2004, 2005, 2006; Pokorny 2000; Pokorny et al. 2009; Sobota et al. 2011; Wieczorek-Dąbrowska et al. 2013; Cappelli et al. 2020; Giżejewska et al. 2020). Antlers are periodically replaced, bony cranial appendages of male deer (and females in the reindeer, *Rangifer tarandus*) that are grown and cast from permanent protuberances of the frontal bones known as pedicles (Goss 1983; Landete-Castillejos et al. 2019). The annual antler cycle of male deer is tightly coupled to their reproductive cycle and controlled by changes in androgen concentrations in blood. In deer from temperate and arctic regions, the reproductive cycle is closely linked to the photoperiod (Goss 1983; Bubenik 1990; Lincoln 1992). Antlers are the fastest forming bones in the animal kingdom and grow during a seasonally fixed timespan of a few months. After the shedding of the skin (velvet) that covers the antlers during their growth, they die off and the bare bony (“hard”) antlers are exposed (Goss 1983; Lincoln 1992; Landete-Castillejos et al. 2019).

Growing antlers can accumulate large amounts of “bone-seeking” elements like lead during their short, seasonally fixed lifespan (Sawicka-Kapusta 1979; Kierdorf and Kierdorf 1999, 2000a, 2000b, 2002a, 2004, 2005, 2006; Pokorny 2000; Pokorny et al. 2009; Sobota et al. 2011; Wieczorek-Dąbrowska et al. 2013; Cappelli et al. 2020; Giżejewska et al. 2020). Antlers can therefore be used to monitor ambient lead levels, as they constitute “naturally standardized” environmental samples (Kierdorf and Kierdorf 1999, 2005, 2006; Tataruch and Kierdorf 2003). In contrast to other bones, the lead content of antlers constitutes a marker of exposure over a medium timescale of a few months (the antler growth period). Furthermore, contrary to other types of bone, the lead content of antlers is not markedly affected by the age of an individual (Pokorny et al. 2004). Since antlers are collected as trophies by hunters and kept in private or public collections, larger sets of antlers with known dates of collection and locality are often available for study. Such antler series constitute environmental archives whose analysis enables the reconstruction of temporal changes in ambient lead concentrations (Kierdorf and Kierdorf 2005, 2006).

Among European cervid species, the European roe deer (*Capreolus capreolus*) is for several reasons particularly suited as a bioindicator. Firstly, it is by far the most abundant deer species, with a harvest (including deaths from other causes than hunting) of 1,226,169 individuals in the hunting year 2019/2020 in Germany, and an annual harvest of more than 1 million animals over the last 20 years (DJV. Deutscher Jagdverband 2021). Secondly, the species is highly adaptable and therefore inhabits a wide range of habitats (Andersen et al. 1998). Thirdly, it has a relatively small home range, enabling a monitoring with rather high spatial resolution (von Raesfeld et al. 1978; Danilkin 1995; Stubbe 1997).

The present paper reports lead concentrations in the antlers of European roe deer that had been culled in a hunting district in Northern Germany over a period of 119 years (1901–2019). This time span covers the period from before the era of mass motorization and the use of leaded gasoline to after the phase-out of leaded gasoline and reduced anthropogenic lead emissions from other sources. We hypothesized that these changes would be reflected by variation in antler lead levels over the study period.

## Materials and methods

### Specimens and study area

We analyzed the antlers of 90 adult roe bucks that had been culled in the hunting district Harsum between 1901 and 2019. All antlers were regenerated ones. Adult roe bucks in Germany cast their old antlers between October and December, followed by antler regrowth during winter. The velvet is shed from the antlers in March/April, and the rutting period extends from mid-July to mid-August (von Raesfeld et al. 1978; Stubbe 1997). Antlers were assigned to the years in which the respective bucks had been taken. For thirteen antlers from the period 1958–1981, the exact year of collection was not known. No antlers from the period 1940–1955 were available, as no hunting was performed in the study area during most of World War II, and private ownership of firearms was not allowed after the end of the war until 1955.

**Fig. 1:**
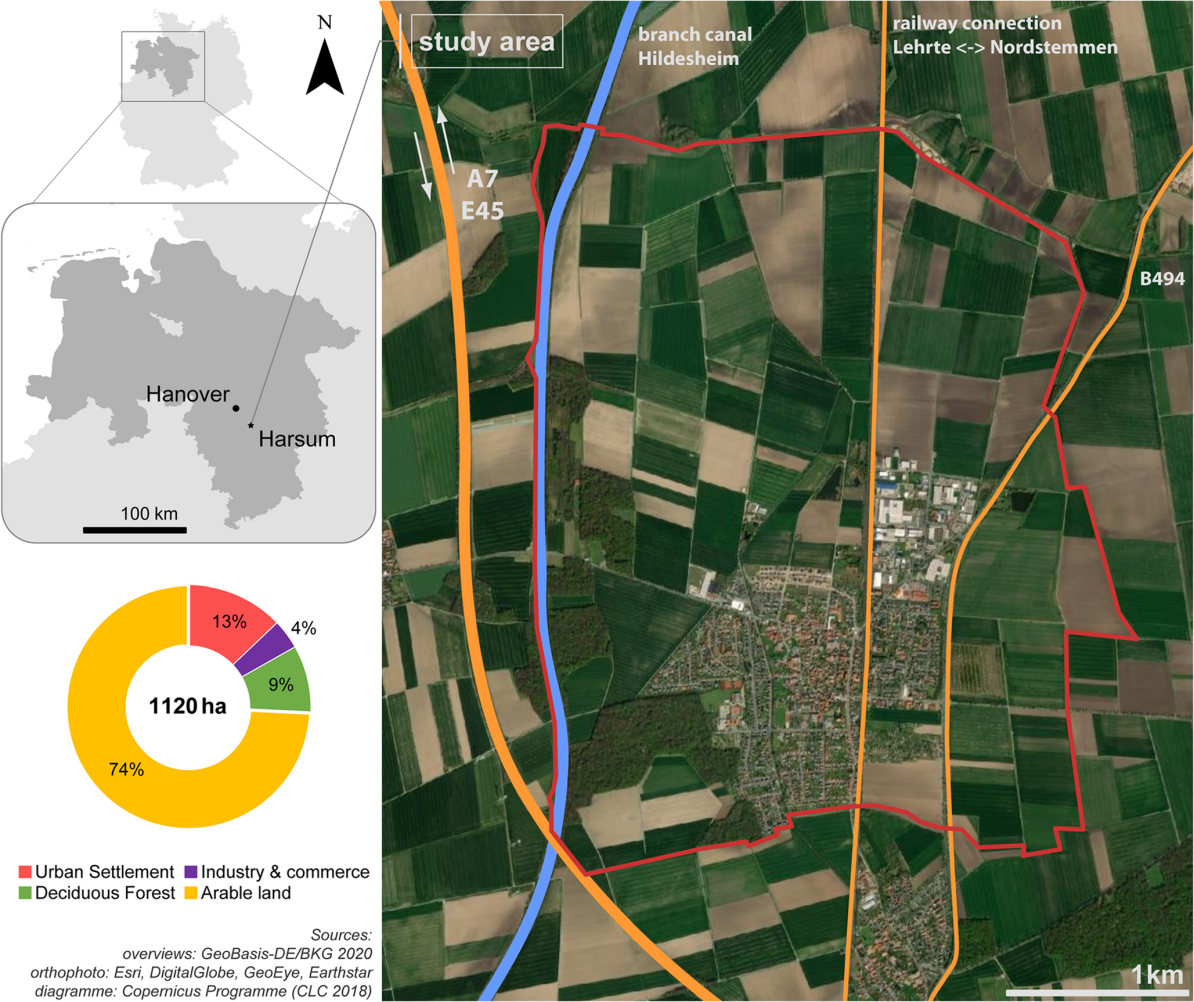
Study area. Localization of the hunting district Harsum in the northern part of the county of Hildesheim, federal state of Lower Saxony, Germany (upper left). Aerial photo of the hunting district Harsum (boundaries are indicated by the red line) (right). The orange lines mark the three major traffic infrastructures, and the blue line indicates the branch canal. Diagram showing the current land use in the study area (lower left)

The hunting district Harsum (altitude about 82 m above sea level) lies in the northern part of the county of Hildesheim (federal state of Lower Saxony, Germany) (Fig. 1). The area has a temperate climate, with annual mean temperature of 8.7°C, an annual precipitation of 676 mm (climate-data.org 2020), and prevailing westerly winds (DWD CDC. Deutscher Wetterdienst Climate Data Center 2018). The hunting district is bordered by a branch canal of the Mittelland canal in the west that started operations in 1928 (Hafenbetriebsgesellschaft mbH Hildesheim n.d.). Less than 500 meters to the west of the hunting district, a north-south oriented section of the motorway A7/E45 is located that was completed in 1962. Further larger traffic infrastructures are the federal highway B494, a section of which is located in the east of the hunting district, and a railway line (electrified in 1965) that divides the district into a larger western and a smaller eastern part. The hunting district has a size of about 1120 hectares and consists mainly of arable farmland (74%) and few patches of deciduous forest (9%). Urban settlements and industrial/commercial areas account for 13% and 4%, respectively (EEA. European Environment Agency 2020). Based on the pattern of land use, the roe deer inhabiting the study area are considered to be of the “field” ecotype (Kałuziński 1982; Demesko et al. 2018). There are nine covered contaminated sites (slurry ponds, dredge material dumps, pond fillings, and a former clay pit) within the hunting district, which add up to an area of approx. 21 ha (2% of the study area; NIBIS Kartenserver 2020). No further information was available about a possible hazardous potential of these areas.

### Bone sampling and analysis

**Fig. 2:**
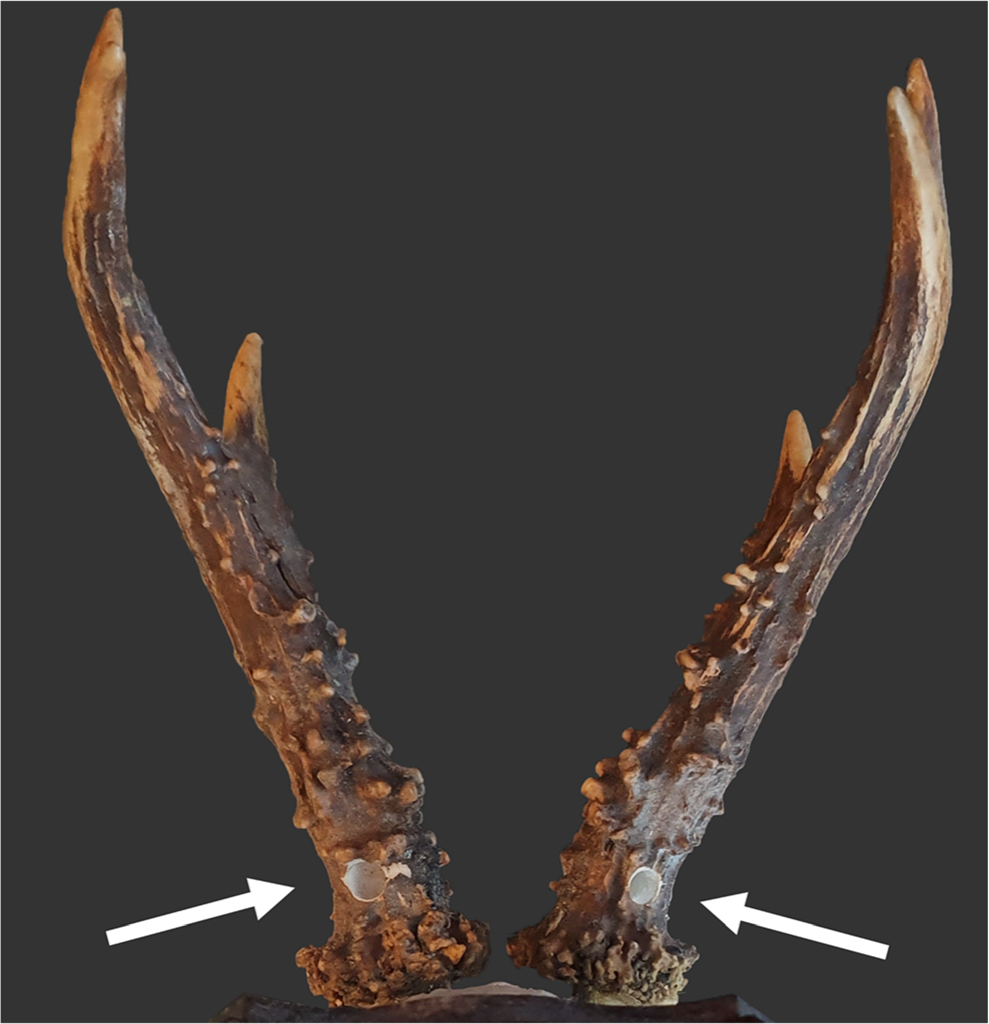
Sampling locations (arrows) at the back of the antlers

Antler bone samples were obtained as described by Kierdorf and Kierdorf (2000b, 2002b). Prior to sampling, the antlers were thoroughly cleaned with a nylon brush to prevent contamination of the bone samples by dust or dirt. Subsequently, a hole was drilled into the back of the main beam of each antler approximately 1.5 cm above the antler-pedicle junction using a hand-held electric drill with a tungsten carbide cutter (Fig. 2). The bone powder obtained from each pair of antlers was collected, thoroughly mixed by stirring, individually stored in a small plastic container, and labeled with a consecutive number for each buck sampled.

Precisely weighed samples (approximately 0.2 g for each double determination) of antler bone powder were digested with 5 ml of 65% (w/v) nitric acid (HNO_3_; Suprapur®) and filtered into glass vials. The digestion vessels were rinsed with high-purity water (AnalaR® NORMAPUR ® ISO 3696 Grade 3), and the content was also filtered into the glass vials, which were made up to 25-ml volume with the high-purity water. Subsequently, each sample was decanted into a 50-ml polyethylene container and stored in a fridge at 4°C until analysis.

Lead concentrations were determined with a graphite furnace atomic absorption spectrometer (ContrAA 800D, Analytik Jena), using a 5-point calibration with 10, 30, 50, 80, and 100 μg Pb/l. After every tenth sample, a certified reference material (NIST Standard Reference Material (SRM)® 1486 Bone Meal) was measured. Recovery rate of the SRM was 96.2 ± 13.4% (mean ± SD), and the nominal concentrations in the antler bone samples were corrected for the recovery rate. The limit of detection (LOD) of the analytical method calculated using the calibration curve method was 0.2 mg Pb/kg. All determined lead concentrations in antler bone, given as milligrams per kilogram on an air-dry weight (d.w.) basis, were ≥ LOD. Individual lead values reported and used for calculation are means of two determinations per bone sample.

### Statistics

All statistics were performed using R version 4.0.3 (R Core Team 2020). Based on the culling years of the roe bucks, antlers were grouped into three sampling periods (period 1: 1901–1939; period 2: 1956–1984; period 3: 1985–2019). Period 1 was a time of rather low motor traffic, with the number of registered motor vehicles in Germany increasing rather slowly from approximately 10,000 in 1906 to 715,000 in 1938 (Hoffmann 2000). Period 2, starting with the permission to again use private firearms for hunting after World War II, was a time of increasing lead emissions due to rapid mass motorization (from 8,003,654 vehicles registered in the FRG in 1960 to 30,617,641 in 1985; KBA. Kraftfahrtbundesamt 2021) and industrial growth. Period 3 started after the introduction of unleaded regular gasoline in October 1984 and covered the time following the ban of leaded gasoline. Differences between the three periods were analyzed by a Kruskal-Wallis H test, followed by pairwise post hoc comparisons using the Wilcoxon-Mann-Whitney U test (two-sided), with Bonferroni adjustments of *P*-values. For all pairwise post hoc comparisons, adjusted *P*-values are reported, and for all statistical tests *P*-values < 0.05 were considered statistically significant.

## Results

The lead content of the analyzed antlers ranged between 0.2 and 10.9 mg/kg. Minimum lead values decreased from period 1 over period 2 to period 3, while the coefficients of variation increased (Table 1).

**Table 1:** Summary statistics of antler lead content (mg/kg, d. w.) from 90 roe bucks (*Capreolus capreolus*) taken in the hunting district Harsum between 1901 and 2019

Period	N	Range	Median (IQR)	Mean (SD)	CV (%)
1 (1901–1939)	39	0.7–10.3	1.6 (1.3–2.0)	1.9 (1.5)	80.2
2 (1956–1984)	19	0.3–8.0	3.2 (2.4–4.6)	3.5 (2.0)	115.9
3 (1985–2019)	32	0.2–10.9	1.3 (0.7–2.4)	2.0 (2.2)	132.0

*IQR*, interquartile range; *SD*, standard deviation; *CV*, coefficient of variation

**Fig. 3:**
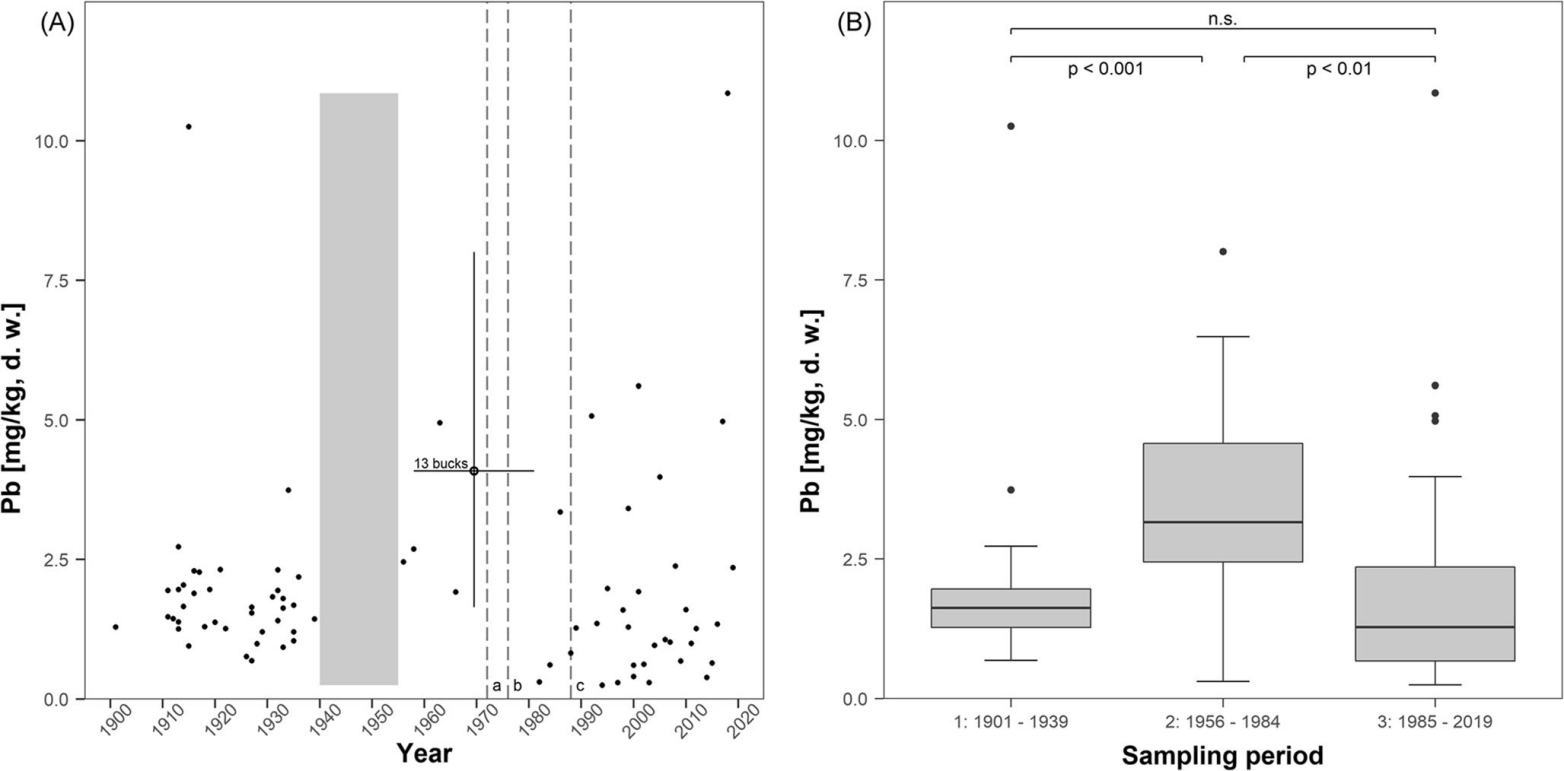
**A** Antler lead content of roe bucks (n = 90) culled between 1901 and 2019 in the hunting district of Harsum, federal state of Lower Saxony, Germany. The cross indicates the ranges of lead values and collection years for 13 antlers whose exact year of collection was not known (bucks culled between 1958 and 1981). The period during which no hunting was performed in the study area is indicated by the gray vertical column. The dashed vertical lines mark the years of important regulations regarding leaded gasoline in Germany: (a) 1972: reduction of lead in gasoline to a maximum of 0.4 g Pb/liter; (b) 1976: reduction of lead addition to 0.15 g Pb/liter; (c) 1988: ban of leaded regular gasoline. **B** Comparison of antler lead content during the three sampling periods, period 1: prior to World War II, period 2: time of high lead emissions from traffic due to the use of leaded gasoline, period 3: following the introduction of unleaded gasoline in the FRG in October 1984. Box-whisker-plots show median (line in box), interquartile range (IQR, box), non-outlier range (Q_1_ −1.5-fold IQR and Q_3_ +1.5-fold IQR, whiskers), and outliers (points)

Antler lead content differed significantly among the three sampling periods (Kruskal-Wallis H test: chi-squared = 15.184, *df* = 2, *P* < 0.001). Concentrations significantly (*P* < 0.001) increased from period 1 (median: 1.6 mg Pb/kg) to period 2 (median: 3.2 mg Pb/kg), and significantly (*P* < 0.01) decreased from the latter to period 3 (median: 1.3 mg Pb/kg*,* Fig. 3). The difference between periods 1 and 3 was not significant (*P* = 0.549). Before World War II, the clustering of the individual values is rather dense, with only one antler showing a markedly increased lead content of 10.3 mg/kg. In period 2, the scatter of the data points is still rather small, with two outlying low values (0.3 and 0.6 mg Pb/kg) of antlers collected, respectively, in 1982 and 1984. Period 3 shows the highest intra-sample variation, as well as the highest individual value (10.9 mg Pb/kg) for an antler collected in 2018.

## Discussion

Our study revealed a marked drop in overall lead concentration in roe deer antlers from the study area following the introduction of unleaded gasoline in the FRG in October 1984. These findings are consistent with the results of other studies that focus on the trend of lead concentrations in antlers of European roe deer over time (Table 2), as well as in antlers of red deer (*Cervus elaphus*) (e.g., Giżejewska et al. 2020) and in other terrestrial biota (e.g., Schnyder et al. 2018; Helander et al. 2019). This indicates the overall success of the phase-out of leaded gasoline and additional measures taken to reduce lead release to the environment from anthropogenic sources.

**Table 2:** Lead concentrations reported for antlers of European roe deer

Country/region^a^	Period/year^b^	N	Lead concentration (mg/kg, d.w.)				Ref.
			Mean	Median	Minimum^c^	Maximum^c^	
Germany/Harsum area	1901–2019	90	2.3	1.6	0.2_(1994)_	10.9_(2018)_	This study
Germany/Cologne area	1932–1998	39	4.2	4.3	0.4_(1996)_	12.0_(1972)_	[1], [2]
Germany/Siegen area	1948–2000	116	7.0	4.1	0.3_(1995)_	166.3_(1967)_	[1], [3]
Germany/Ruhr area	1951–1999	132	5.4	3.4	0.6_(1999)_	19.0_(1960)_	[1], [4]
Germany/Märkischer Kreis	1961–1999	32	8.0	3.8	0.7_(1995/1998)_	58.1_(1964)_	[1], [5]
Germany/13 regions (North Rhine-Westphalia)	1990–1999	172	1.6	1.2	0.3	14.0	[1], [6]
Germany/Northern Hesse (Knüll)	1971–1990	148	8.6–30.4^d^	-	-	-	[1], [7]
Poland/Białowieża Forest	1961–1974	14	3.8	-	-	-	[1], [8]
Poland/Niepołomice Forest	1972–1975	18	4.5	-	-	-	[1], [8]
Poland/Ojców National Park	1968–1973	6	7.6	-	-	-	[1], [8]
Poland/Silesian Forest	1938–1950	17	6.4	-	-	-	[1], [8]
Poland/Silesian Forest	1951–1973	30	14.8	-	3.8	33.3	[1], [8]
Poland/Lower Silesia	1993–1994	23	1.7–2.3^e^	-	0.6	2.9	[1], [9]
Poland/Rogów	1985	32	1.5	-	0.2	3.2	[1], [10]
Poland/Wroclaw	1982–1986	27	20.4	-	-	-	[1], [11]
Poland/Szczecin	2000–2006	11	0.8	-	0.03	2.1	[12]
Poland/Drawsko Pomorskie	2000–2006	9	1.1	-	0.3	1.8	[12]
Slovakia/Sitno	1881–1918	14	25.0	20.7	5.9	75.0	[1], [13]
Slovakia/Sitno	1980–1990	18	3.0	2.2	1.4	9.6	[1], [13]
Slovenia/Šalek Valley,	1961–2002	116	1.3	1.1	0.2_(2000–2002)_	7.3_(1960–1969)_	[14]
Slovenia/Upper Meža Valley	1925–2003	45	54.7	18.1	2.7_(2000–2003)_	554_(1980–1989)_	[15]
Sweden/Bogesund and Garpenberg	1968–1983	28	2.7	-	0.2	8.5	[16]
United Kingdom/Cumbria	1985–1986	10	3.0	-	-	-	[1], [17]

^a^Country and region where the antlers were collected

^b^Collection year or collection period

^c^Year(s) or period of specimen collection given in brackets

^d^Range of twenty means

^e^Range of three means

References: [1] Kierdorf and Kierdorf (2005), [2] Kierdorf and Kierdorf (2000b), [3] Kierdorf and Kierdorf (2004), [4] Kierdorf and Kierdorf (2002a), [5] Kierdorf and Kierdorf (2001), [6] Kierdorf and Kierdorf (2000a), [7] Volmer and Herzog (1995), [8] Sawicka-Kapusta (1979), [9] Chyla et al. (1996), [10] Sawicka-Kapusta et al. (1991), [11] Lorenz et al. (1991), [12] Sobota et al. (2011), [13] Tataruch and Schönhofer (1993), [14] Pokorny et al. (2004), [15] Pokorny et al. (2009), [16] Kardell and Källmann (1986), [17] Samiullah and Jones (1991)

While all other European deer species grow their antlers during spring and summer, antler growth in the European roe deer occurs in autumn and winter. This is an important difference, as lead concentrations in plants browsed by deer are markedly higher in autumn/winter than in spring (Reuter et al. 1996; Pattee and Pain 2003) and therefore roe bucks are exposed to higher lead levels than males of other deer species during the period of antler growth, which is a phase of high mineral demand.

The pronounced variation in antler lead concentration observed in period 3 is of special interest. While the overall decline in lead levels compared to the preceding period can be attributed to a reduction of emission from motor traffic, the increased variation in lead concentrations in period 3 compared to periods 1 and 2 points to an increased variability in exposure conditions of the roe deer from our study area in more recent times. Some bucks apparently took up larger amounts of lead, including the individual with the highest antler lead concentration of the entire sample. Further studies are needed to elucidate the sources and pathways of the underlying high but apparently locally restricted exposure. In previous studies, local point sources could be identified as the cause of an increased pollutant exposure of roe deer (e.g., Kierdorf and Kierdorf 2002b; Pokorny et al. 2009).

Vehicular traffic is today still an important source of lead release to the environment. After the phase-out of leaded gasoline, current lead release from traffic is, however, mainly due to wear of brakes and tires (De Silva et al. 2021). However, due to the persistence and low mobility of inorganic lead, deposition from the previous use of leaded gasoline still contributes to current high lead concentrations in roadside soils (MacKinnon et al. 2011). These authors suggest that previously deposited lead is continuously redistributed, maintaining a more or less constant transfer into the biosphere along roads. In line with this view, also other studies found high concentrations of lead in roadside dust, and in soils and biota along roads (Walraven et al. 2014; Adamiec et al. 2016; Adamiec 2017; De Silva et al. 2021). We assume that roe bucks from our study area were exposed to traffic-related lead near the motorway (A7) and the federal highway (B494). Especially lead bound to the particulate matter fraction of < 2.5 μm in diameter is readily distributed via atmospheric transport and has a high bioavailability (Padoan et al. 2017; De Silva et al. 2021).

It has been stated that deposition on surfaces of preferred feeding plants is a main exposure route of lead for browsing mammals (Tataruch and Kierdorf 2003). According to Demesko et al. (2018), field roe deer exhibit higher lead burdens than conspecifics from forest habitats, as dry deposition of lead on forest floor vegetation is relatively low compared to open fields (Grönholm et al. 2009; Schaubroeck et al. 2014). Regarding the lead exposure of field roe deer, also the contribution of lead from manure, sewage sludge, and mineral fertilizers must be considered (Knappe et al. 2008). It can further be assumed that lead bound to soil particles is mobilized by wind erosion on agricultural fields after harvest, which is also intensified by agricultural tillage, and deposited on grazing plants. In Northern Germany, this is particularly the case in late autumn/winter (Willand et al. 2014), i.e., during the antler growth phase of European roe bucks.

## Conclusion

The present study revealed marked variation in antler lead concentration of roe deer in an agricultural-dominated area of Northern Germany over a period of 119 years. The findings underscore that the analysis of antlers, which constitute “naturally standardized” monitoring samples, provides a suitable tool for assessing temporal variation in environmental lead levels. The widespread, abundant, and highly adaptable European roe deer is particularly suited as a monitoring species in cultural landscapes. A main advantage of using roe deer antlers as monitoring units is that large samples spanning longer periods of time are readily available, thereby allowing the reconstruction of time trends of the level of bone-seeking pollutants in roe deer habitats. As the antlers can be obtained from individuals harvested in the course of regular management operations for population control, there is no need to kill animals only for providing samples. Since antlers are collected and kept as trophies, local hunting communities can provide the necessary material for studies on changing levels of bone-seeking contaminants in their neighborhood. In this way, hunters are able to significantly contribute to environmental surveillance and monitoring in an interesting example of citizen science (Cretois et al. 2020).

## Data Availability

The datasets used and/or analyzed during the current study are available from the corresponding author on reasonable request.
